# Modulation of lipolysis and glycolysis pathways in cancer stem cells changed multipotentiality and differentiation capacity toward endothelial lineage

**DOI:** 10.1186/s13578-019-0293-z

**Published:** 2019-03-27

**Authors:** Ayda Pouyafar, Milad Zadi Heydarabad, Jalal Abdolali Zade, Reza Rahbarghazi, Mehdi Talebi

**Affiliations:** 1 Stem Cell Research Center, Tabriz University of Medical Sciences, Imam Reza St., Daneshgah St., Tabriz, 5166614756 Iran; 2 Drug Applied Research Center, Tabriz University of Medical Sciences, Tabriz, Iran; 3 Department of Applied Cell Sciences, Faculty of Advanced Medical Sciences, Tabriz University of Medical Sciences, Tabriz, Iran

**Keywords:** Glycolysis, Lipolysis, EMT, Ovarian cancer stem cells

## Abstract

Cancer stem cells obtain energy demand through the activation of glycolysis and lipolysis. It seems that the use of approached targeting glycolysis and lipolysis could be an effective strategy for the inhibition of cancer stem cells. In the current experiment, we studied the potential effect of glycolysis and lipolysis inhibition on cancer stem cells differentiation and mesenchymal–epithelial-transition capacity. Cancer stem cells were enriched from human ovarian cells namely SKOV3 by using MACS technique. Cells were exposed to Lonidamine, an inhibitor of glycolysis, and TOFA, a potent inhibitor of lipolysis for 7 days in endothelial differentiation medium; EGM-2 and cell viability was studied by MTT assay. At the respective time point, the transcription level of genes participating in EMT such as *Zeb*-1, -2, *Vimentin*, *Snail*-1, -2 and *VE*-*cadherin* were measured by real-time PCR analysis. Our data noted that the inhibition of lipolysis and glycolysis could decrease cell viability compared to the control of cancer stem cells. The inhibition of glycolysis prohibited the expression of *Zeb*-1, *Snails*, and *Vimentin* while increased endothelial differentiation rate indicated by the expression of *VE*-*cadherin*. In contrast, the inhibition of lipolysis increased EMT associated genes and reduced endothelial differentiation rate by suppressing the transcription of *VE*-*cadherin*. Notably, the simultaneous inhibition of glycolysis and lipolysis had moderate effects on the transcription of EMT genes. We concluded that the modulation of the metabolic pathway of glycolysis in ovarian CSCs is more effective than the inhibition of lipolysis in the control of angiogenesis potential and stemness feature.

Dear editor,

First, we appreciate you for the invitation to provide a response to the Letter-to-the-Editor. Over the past decades, a small fraction of cell populations named cancer stem cells (CSCs) is identified inside the cancer niche, contributing to rapid cancer expansion and resistance to chemotherapeutic agents. In fact, CSCs possess self-renewal, clonogenic and trans-differentiation capacities [1]. Based on the great body of science, CSCs are responsible for the origin, growth, recurrence and cancers metastasis [2]. Thus, any modalities targeting the dynamic growth of CSCs could yield a better therapeutic outcome.

It is found that CSCs change their phenotype via engaging epithelial–mesenchymal transition (EMT) mechanism. These mechanisms adopt CSCs to environmental stimuli and help to escape from the immune-related responses [3]. It is well-established that multiple factors, including ZEB 1, 2, Snail 1, 2, Twist 1, 2, actively participate in the EMT process. The induction of these factors correlates with EMT, contributing to tumor’s invasive behavior and induction of metastasis. Cancer cells, especially CSCs require on-demand energy for dynamics growth, differentiation during the development of tumor mass [4]. Depending on the entity of tumor, cancer cells supply energy requirement by biochemical reactions related to glycolysis, lipolysis, and proteolysis. Similar to the normal cells, cancer cells, notably CSCs, primarily absorb their energy from glycolysis and lipolysis [4]. Based on the previously published data, there is an inevitable relationship between the physiological bioactivity of stem cells and basal energy metabolism [5]. Hexokinase and acetyl-CoA carboxylase (ACC) 1, the main enzymes regulating glycolysis and lipolysis, are at the center of attention and numerous agents were produced to control glycolysis and lipolysis by controlling their bioactivities [6]. As a matter of fact, understanding the close relationship between glycolysis and lipolysis with EMT process could help us to forecast the CSCs behavior and tumor reaction against the distinct chemotherapeutic agent.

In this study, we isolated CSCs from the human cell line; SKOV3. To analyze the potential effect of glycolysis and lipolysis inhibition on the EMT of CSCs, we treated these cells with Lonidamine and TOFA and the expression of the key genes participating in EMT was monitored. In addition, endothelial differentiation of CSCs was evaluated by the expression of endothelial specific marker VE-cadherin under the inhibition of lipolysis and glycolysis. It seems that the results of the current experiment could shed a light on the potential role of glycolysis and lipolysis in the ability of CSCs to maintaining stemness feature and commitment toward mature cell type.

CSCs were enriched by using magnetic activated cell sorting via CD133 microbeads (Order no: 130-097-049; Miltenyi Biotec; Germany). Identification of SKOV3 cell line CSCs was performed by flow cytometry and immunofluorescence analyses (Fig. 1a, b). Monitoring the protein level of stemness-related markers in isolated CSCs by IF imaging revealed the FITC-conjugated CD133 cells (Fig. 1a). Similar to this panel, flow cytometry confirmed that more than 96% (96.9 ± 2.4%) of isolated cells expressed CD133 (Fig. 1b).

**Fig. 1:**
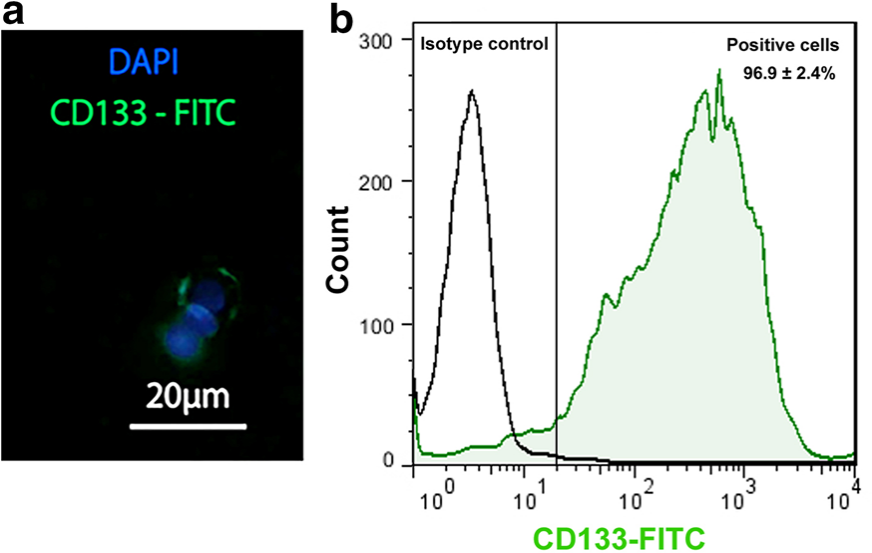
Characterization of isolated CSCs by immunofluorescence and flow cytometry analyses. Data confirmed the existence of CD133 indicated by positive color cells by immunofluorescence imaging (**a**). Flow cytometry showed that more than 96% of cells were positive for CD133 (**b**). Data are expressed as mean ± SD. Blue = DAPI; Green = FITC-conjugated CD133

## Combination of TOFA and Lonidamine diminished CSCs survival rate

**Fig. 2:**
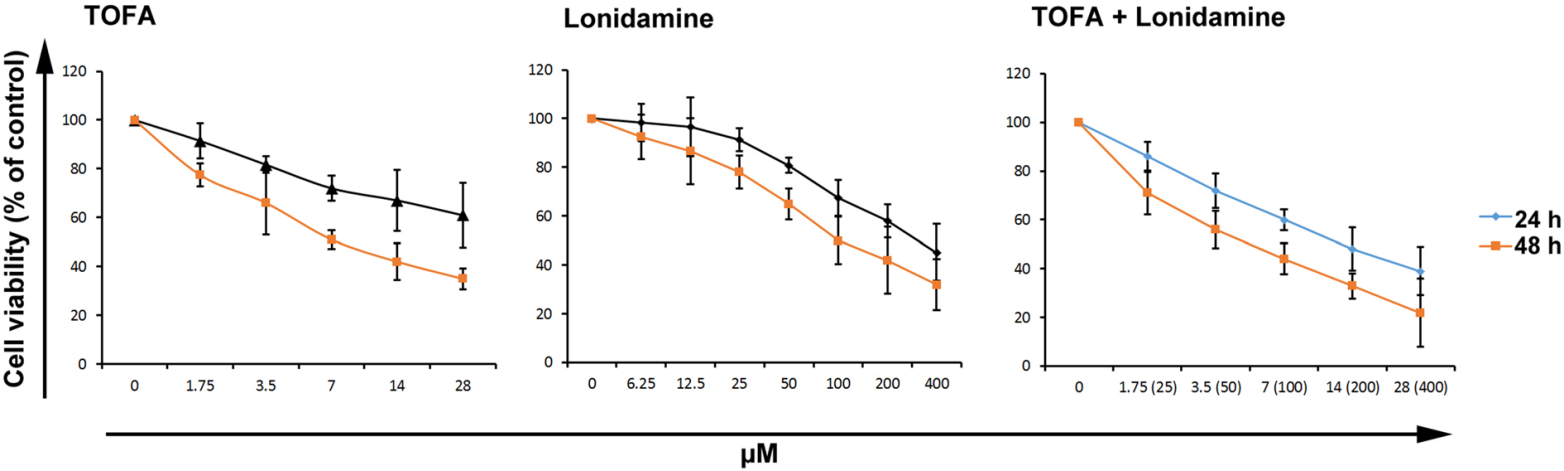
CSCs survival assay. MTT assay revealed the detrimental effects of TOFA and Lonidamine in a dose and time-dependent manner. The combination of TOFA with Lonidamine showed synergistic cytotoxicity on CSCs in a dose and time-dependent manner (n = 3). Data are shown as mean ± SD

We incubated the CSCs with Lonidamine (Cat no: L4900 Sigma-Aldrich) and TOFA (Cat no: T6575, Sigma-Aldrich) and explored the role of glycolysis and lipolysis on CSCs viability. We found that CSCs survival rate was reduced by increasing the concentration of Lonidamine and TOFA after 24 and 48 h (Fig. 2). At time points 24 and 48 h, by increasing the concentration of Lonidamine from 50 to 100 μM, CSCs mortality was enhanced from 50 to 65% after 24 h and reached to 67 to 80% at time point 48. In TOFA-treated cells, the increase of drug from 3.5 to 7 μM resulted in elevated cell death from 51 to 66% at time point 24 and 72 to 81% after 48 h (Fig. 2). Based on the data, CSCs treated with a combination of TOFA and Lonidamine, a higher cytotoxic effect was found when compared to CSCs treated either with TOFA or Lonidamine alone (Fig. 2).

## CSCs treatment with lipolysis and glycolysis inhibitors modulated gene expression related to angiogenesis and EMT

**Fig. 3:**
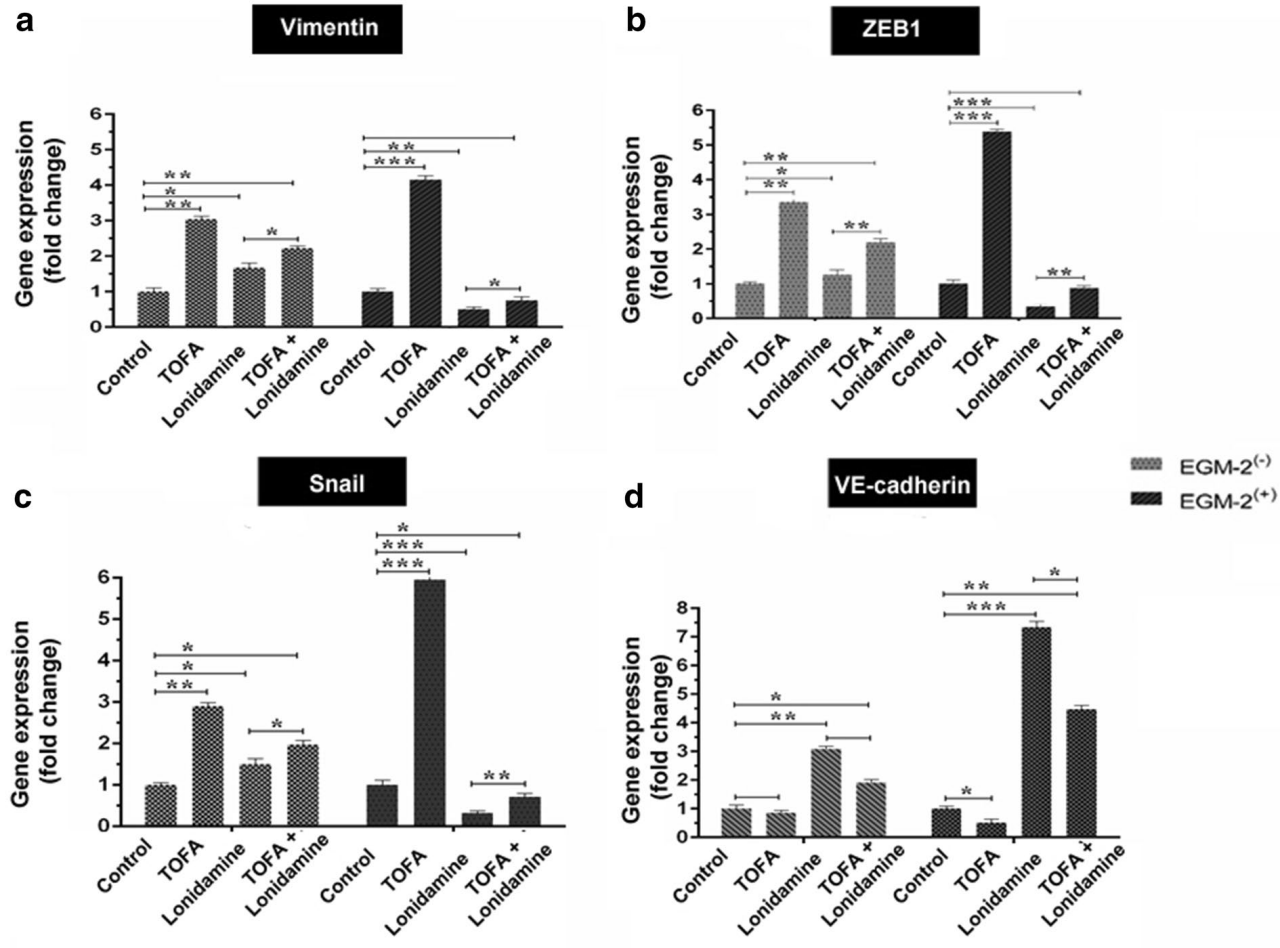
The real-time PCR analysis of EMT-associated genes in CSCs from HT-29 cells. Data are expressed as mean ± SD. One-way ANOVA and Tukey (post hoc) analysis (n = 3). *p < 0.05; **p < 0.01; ***p < 0.001

Gene expression assay was performed to evaluate the effect of glycolysis and lipolysis inhibition on the expression of key genes associated with EMT such as *ZEB1*, *vimentin*, *Snail* (Table 1). We also monitored the transcription level of *VE*-*cadherin* as an endothelial marker (Fig. 3a–d). Data showed that the incubation of CSCs with 7 μM TOFA for 7 days caused a significant increase in the expression of genes *ZEB1*, *Vimentin*, *Snail* coincided with the down-regulation of *VE*-*cadherin* gene in CSCs incubated in endothelial growth medium and normal condition compared to the control-matched CSCs (*p *< 0.05; Fig. 3a–d). Our data showed the superior effect of TOFA in CSCs from EGM-2 compared to normal medium (Fig. 3). Based on our data, incubation of treated CSCs with 100 μM Lonidamine inhibited the transcription of genes participated in stemness indicated by down-regulation of *ZEB1*, *Vimentin*, *Snail* while the mRNA content of VE-cadherin increased after 7 days (*p *< 0.05; Fig. 3a–d). It seems that changes were more evident in differentiating CSCs toward endothelial lineage. In a better word, incubation of CSCs with endothelial growth medium supplemented with Lonidamine decreased stemness feature and decrease progenitor cells. Our results showed the simultaneous treatment of CSCs with TOFA and Lonidamine found to be intermediate in the induction and/or inhibition of EMT. Results showed that Lonidamine alone had more effect on the inhibition of EMT compared to the combined regime of Lonidamine and TOFA, affecting the expression of *ZEB1*, *vimentin* and *Snail* genes in vitro.

**Table 1:** Primer sequences used for real-time PCR

Gene	Sequences (5′-3′)	Annealing Temp (^o^C)	Amplicon length (bp)
Vimentin	F: CAGATTGGCTGAAATGGATGAGAA R: TAGGTGGCGATGTCAATGTCAA	62	173
ZEB1	F: CTGGAGAAAAGCCCTATCAATGT R: CTGTCAACATCCTGGTCCCTTGT	62	244
Snail	F: GAGTTTAAATTCCAGCTGCC R: CAGAGTCCCAGATGAGCATT	62	109
VE-cadherin	F: TGAAGAATTACGAGTCGGAC R: CACAGTCGGATGTGTCGGTGC	65	129
18S RNA	F: TCGGCTGGAGAAGAGCTACG R- GTACTTTCGTGGATGTTACA	57	131

Here, we inhibited glycolysis and lipolysis and hypothesized that this strategy could reduce EMT and endothelial differentiation of CSCs. Based on data, we found that the suppression of glycolysis and lipolysis by Lonidamine and TOFA could reduce CSCs survival rate. As expected, the simultaneous suppression of glycolysis and lipolysis had a more cytotoxic impact on HT-29 line CSCs. The emergence of CSCs is induced under the hypoxic condition and many tumor cells and CSCs shift their basal metabolism to anaerobic respiration. It is likely to mention that the use of Lonidamine could cease the glycolysis reaction in hypoxic tumor cells and CSCs, decrease energy supply and sensitize them to chemotherapy. Consistent with the current experiment, the inhibition of glycolytic anaerobic metabolism in CSCs from a human osteosarcoma cell line contributed to cell death [7]. Similar to glycolysis inhibition, we found that the reduction of lipolysis affected CSCs survival. It was shown that lipid metabolism not only participates in energy production but triggers biochemical pathways of redox homeostasis [8].

According to our data, Lonidamine reduced the expression of genes maintaining stemness feature and increased endothelial differentiation of CSCs. The reversed effects were found in CSCs exposed to TOFA. One could hypothesize that lipolysis inhibition alone cannot be an interesting therapeutic approach to cease the vasculogenic capacity of CSCs. The alteration of glycolysis seems to be a more effective strategy to reduce the acquisition of stemness in tumor niche compared to the modulation of lipolysis. Consistent with our data, Zhao and colleagues found that the increase of glycolysis rate induces stemness feature and epithelial-to-mesenchymal transition gemcitabine-resistant pancreatic cancer cells [9]. The low level of reactive oxygen species is controlled by the progression of glycolysis and glucose metabolism and correlates with stemness acquisition [10]. Disruption of reactive oxygen species regulation causes serious adverse effects on CSCs. It seems that the promotion of CSCs commitment to other lineage contributes to the control of tumor cells metastasis. In an experiment, treatment of colon cancer cells (cell line SW480) with a tumor suppressor Abhd5, reduced cell metastasis while increased the transcription of VE-cadherin. In a study conducted by Garcia et al., they found that inhibition of lipolysis with TOFA aborted acyl CoA carboxylase activity and increased the intracellular content of acetyl CoA, induced factor SMAD2 and increased stemness feature. We concluded that lipolysis inhibition is not as effective as glycolysis inhibition to prevent the differentiation of CSCs into endothelial-like. It seems that the suppression of glycolysis is more effective to decrease the CSCs population and thereby reduce tumor resistance to chemotherapeutic agents. Studies on the inhibitory effect of glycolysis and amino acid metabolisms and lipolysis under normal and hypoxic conditions could give us more data on the biology of CSCs and tumor resistance to therapies.
