# Correction to: Modulation of lipolysis and glycolysis pathways in cancer stem cells changed multipotentiality and differentiation capacity toward endothelial lineage

**DOI:** 10.1186/s13578-019-0301-3

**Published:** 2019-05-09

**Authors:** Ayda Pouyafar, Milad Zadi Heydarabad, Jalal Abdolalizadeh, Reza Rahbarghazi, Mehdi Talebi

**Affiliations:** 1 Stem Cell Research Center, Tabriz University of Medical Sciences, Imam Reza St., Daneshgah St., Tabriz, 5166614756 Iran; 2 Drug Applied Research Center, Tabriz University of Medical Sciences, Tabriz, Iran; 3 Department of Applied Cell Sciences, Faculty of Advanced Medical Sciences, Tabriz University of Medical Sciences, Tabriz, Iran

## Correction to: Cell Biosci (2019) 9:30 10.1186/s13578-019-0293-z

In the publication of this article [1], there is an error in one of the contributing author names.

The error: ‘Jalal Abdolali Zade’

Should instead read: ‘Jalal Abdolalizadeh’

This has now been updated in the original article [1].
